# Routing in Mobile Wireless Sensor Networks: A Leader-Based Approach †

**DOI:** 10.3390/s17071587

**Published:** 2017-07-07

**Authors:** Unai Burgos, Ugaitz Amozarrain, Carlos Gómez-Calzado, Alberto Lafuente

**Affiliations:** 1 University of the Basque Country UPV/EHU, Barrio Sarriena, s/n, 48940 Leioa, Vizcaya, Spain; unai.burgos@ehu.eus (U.B.); ugaitz.amozarrain@ehu.eus (U.A.); 2 Wimbi Technologies S.L., 20015 Donostia, Spain; unai.burgos@wimbitek.com (U.B.); carlos.gomez@wimbitek.com (C.G.-C.)

**Keywords:** mobile wireless sensor networks, routing, leader election

## Abstract

This paper presents a leader-based approach to routing in Mobile Wireless Sensor Networks (MWSN). Using local information from neighbour nodes, a leader election mechanism maintains a spanning tree in order to provide the necessary adaptations for efficient routing upon the connectivity changes resulting from the mobility of sensors or sink nodes. We present two protocols following the leader election approach, which have been implemented using Castalia and OMNeT++. The protocols have been evaluated, besides other reference MWSN routing protocols, to analyse the impact of network size and node velocity on performance, which has demonstrated the validity of our approach.

## 1. Introduction

Wireless Sensor Networks (WSNs) [1] are used for monitoring and data gathering from the physical world in many applications, such as environment monitoring, farming management, tracking animals or goods, health care, transportation and ubiquitous home networks. Nowadays, WSNs are attracting great attention in research (for a recent survey, see, for example, [2]).

A WSN consists of a usually big number of *sensor nodes*, also called *motes*, deployed in the application scenario. Motes are equipped with the specific sensors demanded by the application, and gather information about the environment, which is transmitted towards one or more *sink nodes* (also called base stations). Sink nodes collect and process the received data in order to make it available to the user. Although in a small WSN one-hop communication to the sink can be implemented, in general, a multi-hop schema must be considered. In this case, regular motes are in charge of executing a routing protocol in order to forward the information towards the sink.

Since motes must usually operate unattended for a long time, they have severe energy constraints. This has a deep impact on the design of a WSN and specifically on the routing protocol. Since communication is a costly resource in terms of energy consumption, a brute-force message forwarding mechanism (i.e., flooding) is in general inadvisable. Instead, the design of the routing protocol [3] is a critical aspect that should consider trade-offs between transmission power and forwarding strategies in order to provide reliability and quality of service. Additionally, since a mote can crash due to battery exhaustion or other reasons, an efficient routing protocol should be flexible enough to react to a failure by reconfiguring the network [4].

Traditional application scenarios of WSN include *static* deployments. In a static WSN, routing can be initially configured to optimise communication and reduce latencies. However, some particular nodes, specifically the ones closer to a sink are especially active in message forwarding, which results in the drop of battery level, and henceforth in a higher probability of message loss due to the reduction of its communication range. Thus, even in static WSN, maintaining an efficient level of operation requires routing reconfiguration. This can be done on the basis of the information about the *quality of links* [5].

As technology matures, WSN are gaining opportunities for application in *mobile* deployments [6]. There are two ways to introduce mobility in a Mobile WSN (MWSN). The simpler one is to have static sensor nodes while the sink nodes are the ones moving. For example, crops on a farm may have sensors that take measurement about the humidity or temperature and whenever a farmer walks by, his smartphone acts a sink node in order to download that information. The second approach is to maintain static sinks while sensor nodes are mobile, for example, when attached to animals in tracking applications. In this case, a static sink can be used to collect tracking information stored in the sensor nodes when the animals are in its range. Finally, both approaches can be combined, letting all nodes in the WSN be mobile. For example, in a residential environment for aged people or for people with disabilities, sensors attached to them can provide information to the mobile devices of the assistant personnel [7].

Despite the additional complexity of routing protocols for MWSN, mobility brings the opportunity of reducing the number of hops to the sink node. According to [8], the probability of having at least a sensor node in the range of a sink increases with the communication radius, the velocity of the node and the number of sinks, resulting in a reduction of the latency.

However, high mobility conditions could prevent many transmissions to successfully deliver messages [9]. On the one hand, any wireless communication requires a minimum time to be completed. On the other hand, a more complex routing protocol could require additional stability conditions for a sensor node to communicate with the sink [10].

This paper, which extends a preliminary work of ours [11], presents a routing approach for Mobile Wireless Sensor Networks. The approach is inspired on leader-election techniques for dynamic systems that we have explored previously [9,12]. It uses a measure of the link quality to maintain a reconfigurable spanning tree in the network graph in order to minimize the transmission cost from the mobile sensor nodes to the mobile sink while providing a high message delivery rate. We present two alternative protocols which implement this approach. The first one (LBR1) uses a reactive, asynchronous forwarding mechanism to propagate leader election messages, while the second protocol (LBR2) is based on a periodic communication pattern.

We also present an evaluation of the protocols, basically on the basis of the message delivery rate, regarding their scalability and their resilience to mobility.

Approaches based on the use of a reconfigurable spanning tree have been proposed before [13]. However, as far as we know, our proposal is the first one for MWSN that uses leader election as a basis for the reconfiguration.

The rest of the paper is organised as follows. In Section 2, we review some works related to ours. Section 3 describes our approach and the two routing protocols that implement it. Section 4 presents the evaluation environment and the performance results of our protocols in comparison with two other related ones. Finally, Section 5 is devoted to conclude the work and present future research directions.

## 2. Related Work

In this section, we revise some routing techniques that have been developed in recent years, specifically those that can be applied to MWSN.

Many works address sink mobility; however, only a few consider the mobility of sensors nodes. These works are the ones addressed in this section. Furthermore, we limit this section to proactive protocols, a feature of ours.

In order to avoid flooding and be energy efficient, hierarchical routing has been adopted in many works. For static WSNs, Heinzelman et al. proposed the Low-Energy Adaptive Clustering Hierarchy (LEACH) protocol [14], which defines a set of clusters in the network. In every cluster, a Cluster Head (CH) is in charge of aggregating the information of the cluster’s nodes and route it towards the sink. In a variant of this protocol [15], a hierarchy of clusters is used to increase scalability.

In MWSN, the cluster approach is followed in the Enhanced Cluster-Based Routing protocol (ECBR-MWSN) [16], which considers mobile sensor nodes; however, it assumes a static sink. The CH is chosen on the basis of a combination of the highest residual energy, lowest mobility and least distance from the sink. To face mobility, ECBR-MWSN elects a new CH periodically, which results in new paths to the sink. Although we do not follow the same cluster-based approach as ECBR-MWSN, our protocols perform periodic casts of the topology too.

In the protocol proposed in [17], nodes cooperate with each other with the objective of enhancing the robustness of routing while using Wireless Broadcast Advantage (WBA) [18], as we also do. In short, WBA refers to the fact that when a node sends a broadcast message all the nodes that are in its transmission range will receive the message. They also assume that every node in the network has a pre-established path between the node and the sink. Special nodes, called *guard nodes*, are also used to support routing of the messages.

Proactive routing, used in early, static WSNs (e.g., the Destination-Sequenced Distance Vector protocol, DSDV [14,19]), has been implemented in [16,17] for MWSN. A proactive routing protocol runs independently from the application to maintain and update routing tables, with the aim of reducing the end-to-end delay, a relevant Quality of Service (QoS) parameter.

As mentioned before, Crowcroft et al. [13] uses a reconfigurable spanning tree. Once the spanning tree has been built, they apply a simple leader election mechanism to set the root of the tree. Contrary to this, in our approach leader election is the basis for the spanning tree construction.

Ad-hoc On-Demand Distance Vector (AODV) protocol [20] is a classical on-demand routing protocol originally designed for Mobile Ad-hoc Networks (MANETs) that has been taken as a reference in many works. In AODV, a node floods route-request messages and waits for replay messages in order to update its routing tables. Additional periodic messages are used to detect disconnections.

Some routing protocols avoid collisions by strongly relying on the Media Access Control MAC layer mechanisms, as it is the case of the Robust Ad-hoc Sensor Routing RASeR protocol [21], which uses Global Time Division Multiple Access (GTDMA). This requires perfect synchronization and a static membership in the set of nodes. In common with our approach, RASeR uses the number of hops as a criteria for routing.

In a previous work [11], we presented a leader election based protocol to build and maintain a spanning tree. This protocol is a preliminary implementation of the approach we define in Section 3. The preliminary version was reactive, like LBR1, and it included handshaking in communications. In our current protocols, we have removed handshaking. Furthermore, as we have commented before, LBR2 is not reactive.

In Section 4, we compare LBR1 and LBR2 to AODV and RASeR.

## 3. A Leader-Based Routing Approach (LBR)

In this section, we describe our approach to route messages in MWSN, which includes the construction and maintaining of the spanning tree (described in Section 3.2) that is used for the routing of application data (described in Section 3.3).

The construction and maintaining of the spanning tree, which is the core of our approach, is based on dynamic leader election, commonly studied in the field of fault tolerant distributed agreement [12,22,23]. We present two alternative protocols to implement this approach. The first one (Subsection 3.2.1) uses a reactive, asynchronous forwarding mechanism to propagate leader election messages. Differently, the second protocol (Subsection 3.2.2) piggybacks the messages on periodic heartbeats messages.

### 3.1. Notation and Assumptions

We consider an architecture with an unknown set of *n* resource-constrained mobile sensor nodes and one more powerful mobile sink. We denote this set of sensor nodes as $V=\{p_{1},p_{2},\ldots ,p_{n}\}$. When required for simplicity, we will also refer to a node as *p*, *q*, etc. We will denote as *s* the sink node. Every node of the system has a unique identifier and a local clock to measure real-time intervals.

Every node, including the sink node, can move. The mobility of a node is characterized according to two dimensions: velocity and direction. At any time *t*, a node *p* moves towards some direction d(p,t) with a velocity $vl\left(p,t\right)\leq VL_{\max}$, $VL_{\max}$ being the maximum velocity of every node *p* in the system.

A node can leave the system, due to a fault or disconnection, or join the system. Connections and disconnections can be caused by mobility, as the node enters and comes out of the communication range of other nodes in the system, or for other reasons. In the following, we formalise this concept.

Every node uses broadcast communication to exploit the Wireless Broadcast Advantage (WBA) [18]. At any time *t*, a node *p* can communicate directly (i.e., at one hop) with a subset $N_{p}\left(t\right)\subset V$, which are the nodes that are at time *t* into a radius *r* from *p*, also referred as the neighbourhood of *p*. The communication range *r* is a common parameter for all nodes in *V*. For simplicity, we consider that message transmission delays are bounded.

We define the parameter *link quality* as a measure of the transmission power (in dBm), which can be easily extracted in a node *p* at the reception of a message by *p* from *q*. We will use the notation $W_{p\leftarrow q}\left(t\right)$ to refer to the link quality for a link (q,p) at time *t*. We assume that $W_{p\leftarrow q}\left(t\right)=W_{q\leftarrow p}\left(t\right)$. We define ξ(tech) as the threshold for the RSSI (Received Signal Strength Indicator) such that a link can be considered to have good link quality.

When considering link quality, a graph G(t) representing the connectivity conditions in *V* at time *t* is obtained. We also define a spanning tree T(t)⊂G(t). Ideally, T(t) will include only links with good link quality.

Observe that partitions in G(t) are possible, but we assume that ∀t, s∈T(t). A node *p* can be disconnected from T(t) due to either: (a) $p\notin N_{q}\left(t\right)$∀q∈T(t), or (b) $W_{p\leftarrow q}\left(t\right)\leq \xi \left(tech\right)$ ∀q∈T(t).

### 3.2. Building the Communication Tree

The goal of our approach is to provide an efficient communication route from any node *p* to the root node *s* in scenarios where sensor devices are mobile. To do so, our approach creates a spanning tree T(t) with the root in the sink node *s*. The spanning tree is continuously maintained in order to represent the best routing options at time *t* on the basis of the link quality information, providing a foundation for efficient routing by maximising the probabilities of successful communications, and henceforth increasing message delivery rates and reducing communication costs.

**Figure 1:**
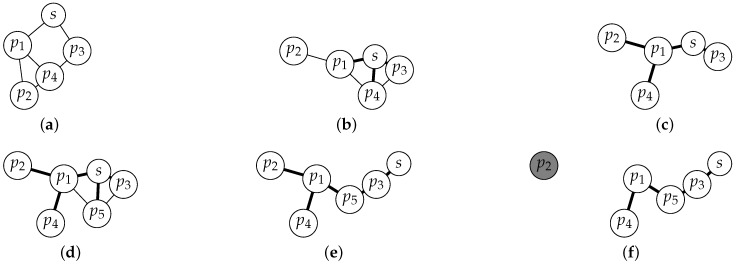
Example sequence illustrating how a spanning tree is built by our algorithms. Solid lines represent spanning tree arcs. (**a**) the sink node *s* starts executing the protocol; (**b**) the spanning tree is built from *s*; (**c**) the spanning tree is complete. Observe that it has been reconfigured in the meanwhile; (**d**) a new node, $p_{5}$, joins the network; (**e**) a new reconfiguration due to mobility; and (**f**) node $p_{2}$ disconnects from $p_{1}$ and becomes not connected.

Figure 1a–f illustrate how a spanning tree is built in situations of node movement, the arrival of a new node (Figure 1d), or a disconnection (Figure 1f).

We now describe the algorithmic details of the approach. First, we focus on the common features of the two alternative protocols we propose in this work. Then, we devote two specific subsections to describe the different mechanisms used in each of the protocols. Please refer, for example, to the first of the protocols (Algorithms 1 and 2) to check the details of the common description.

Both protocols are based on a broadcast primitive. Note that, apart from providing energy-efficiency in the multi-hop information dissemination [18], broadcast is also useful to detect changes in the neighbourhood.

The sink node *s* is in charge of generating new rounds in the spanning tree reconfiguration. The spanning tree is created from the sink node *s*, which is the root of the tree, to the rest of nodes. In this regard, *s* sends leader messages with refreshed round numbers each β time (see Algorithm 2). Every node $p_{i}$ is continuously listening to the communication channel.

Every node $p_{i}\in V$ proposes its leader, denoted $sinkBy_{i}$. Leader election is executed at every node $p_{i}$ at time *t* such that $sinkBy_{i}\in N_{p_{i}}\left(t\right)$ and has the minimum distance to *s* in the spanning tree T(t). Ties are broken on the basis of the node identifier.

The explicit perception that a node $p_{i}$ has about its connection with *s* is another common feature of both protocols. This perception is represented by the variable $active_{i}$, which is True when $p_{i}$ is connected. This explicit knowledge of connectivity is provided by the monitorization of the sink node *s* using the timer $timer\_connectivity_{i}$. Each time a node $p_{i}$ receives a new round identifier, $p_{i}$ re-sets again the timer, denoting $p_{i}$ as connected. Otherwise, if $timer\_connectivity_{i}$ expires, $p_{i}$ will consider itself as not connected.

Observe that, while the round identifiers created by *s* lower-bounds the interval between two consecutive reconfigurations, $timer\_connectivity_{i}$ upper-bounds the interval between two consecutive reconfigurations.

In the following subsections, we describe the two implementations of our leader-based routing (LBR) approach, namely the reactive, eager version (LBR1) and the synchronous, lazy version (LBR2).

#### 3.2.1. Protocol LBR1

Our first implementation relies on delivering the routing information as soon as possible in a reactive way. In this regard, a node $p_{i}$ computes the $sinkBy_{i}$ election during a whole round. When a message with a higher round number is received by a node $p_{i}$, $p_{i}$ broadcasts a leader message with the result of the previous round computation and the increase of the distance received (The distance is extracted from contextual information stored in the message.) (see Lines 5–11, of Algorithm 1). Thus, $p_{i}$ sends its own leader message using the last distance to *s* known by $p_{i}$. Observe in Algorithm 2 that no other node but *s* will send in its leader message a zero distance value.

These new leader messages sent by $p_{i}$ is essential for multi-hop scenarios. First, leader messages propagate the existence of a sink node in the system. Second, the periodic broadcast of the leader message allows for other nodes to become connected. Finally, leader messages provide the required information to its neighbourhood to create the spanning tree. Consequently, the reception of a new round identifier from *s* by a node $p_{i}$ enables a new election round in every $p_{i}$, which eventually results in a reconfiguration of the spanning tree.

**Algorithm 1:** The algorithm LBR1 executed by any sensing node $p_{i}$. Lines 26–38 are also shared with LBR2.
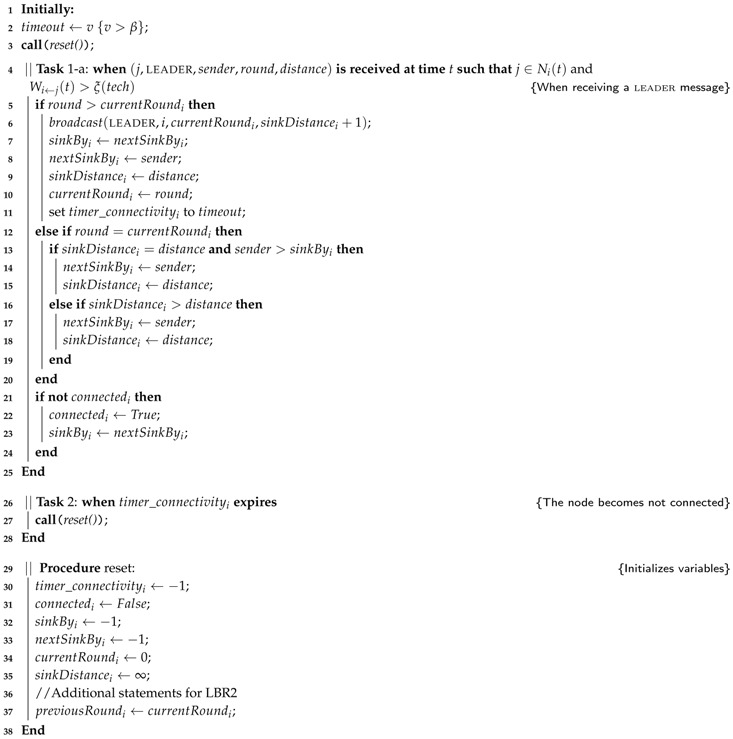


**Algorithm 2:** Algorithm executed by the sink node *s*.
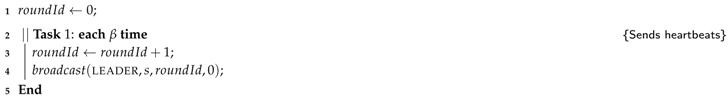


The fact that the sink node broadcasts leader messages periodically (Algorithm 2) allows for a timer-based monitoring of connectivity changes. The first time that a node $p_{i}$ receives a leader message (let us say, at time *t*) from any $p_{j}\in N_{p_{i}}\left(t\right)$, $p_{i}$ becomes connected and will remain connected during the time interval (t,t+timeout] (The timeout variable has a common value slightly higher than β in order to prevent premature time outs.) by setting the timer $timer\_connectivity_{i}$ by Line 11. In this way, every $p_{i}\in N_{s}\left(t\right)$ will become connected by Lines 21–24 and will change their variable $sinkBy_{i}$ pointing to *s* (Lines 7 and 8). If $p_{i}$ does not receive a new round identifier timely, then, either $p_{i}$ will shortly receive a new message by any other path, setting again $timer\_connectivity_{i}$ by Line 11 and reconfiguring the spanning tree, or, otherwise, $p_{i}$ is no longer connected to the spanning tree T(t). In the last situation, the no reception of a new round identifier will cause $timer\_connectivity_{i}$ to expire and $p_{i}$ will become not connected. Thus, the $timer\_connectivity_{i}$ variable represents the uncertainty of $p_{i}$ to be connected to *s* and the expiration of $timer\_connectivity_{i}$ results in $p_{i}$ not connected (Lines 26 and 28). Figure 1e,f illustrate this situation for node $p_{2}$.

#### 3.2.2. Protocol LBR2

Observe than in our second protocol version, LBR2 (Algorithm 3), the broadcast at Line 6 of LBR1 in Figure 1 has been removed. Instead, in LBR2, we introduce a task executed in every node $p_{i}$ to broadcast every α time the current routing information of $p_{i}$ when $p_{i}$ considers itself as connected (see Lines 30–34 in Algorithm 3). This allows us to adopt a windowed mechanism at the MAC layer and reduce the probability of collision in communications.

**Algorithm 3:** The modifications required by LBR2 to the previous algorithm to be executed by any sensing node $p_{i}$.
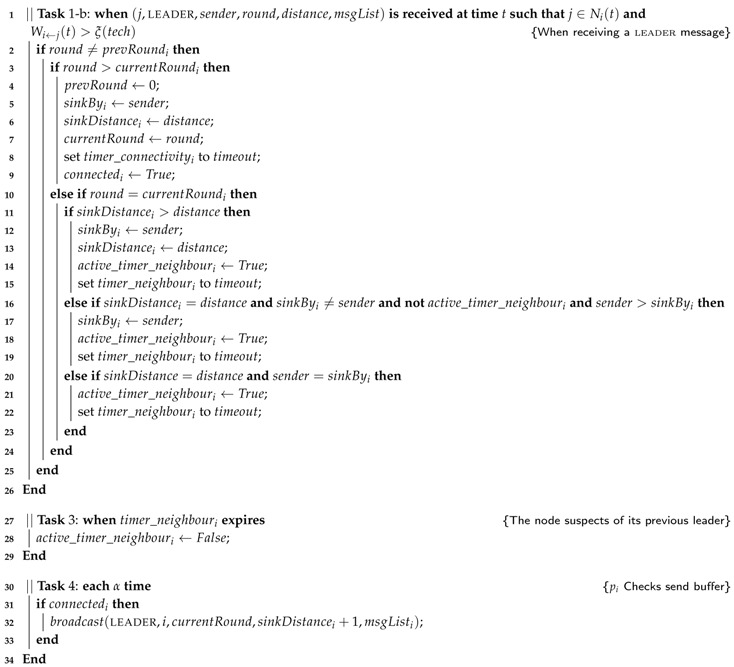


Additionally, in this second protocol, we modify the leadership criteria as follows. The $sinkBy_{i}$ node will be the one with the lowest distance to *s* that firstly communicates with $p_{i}$. This mechanism is a novel strategy that allows balancing the energy consumption in intermediate nodes at routing time. If many of the nodes share the same $sinkBy_{i}$, this leader will eventually saturate and the system performance will decrease in terms of battery. To correctly load balance the spanning tree, in LBR2, we introduce an extra timer to monitor the current parent node. This $timer\_neighbour_{i}$ is set when a new $sinkBy_{i}$ node is adopted by $p_{i}$ (Line 15 in Algorithm 3). Since every node $p_{i}$ sends its information periodically by Line 32, $sinkBy_{i}$, it will also send a leader message resetting again the timer_neighbour by Line 22. On the other hand, if $timer\_neighbour_{i}$ expires (Lines 27–29), $p_{i}$ is allowed to re-elect another $sinkBy_{i}$ among those that have the same distance to *s*, i.e., the condition in Line 16 is satisfied since $active\_timer\_neighbour_{i}$ has been previously set to false by Line 28.

Note that the higher β and timeout values are, the slower the graph connectivity change detection is. This introduces a trade-off between the reaction time and the number of messages sent by the system in order to provide good QoS and energy-efficiency at the same time.

Observe also that the global number of messages sent is linear with the number of nodes in the system, which makes LBR2 scalable regarding communication cost.

### 3.3. Data Routing Protocol

Once an initial spanning-tree has been created, an application-data routing protocol is executed. In contrast to the spanning-tree creation, where we use broadcast as the communication primitive, for application data, we use one-to-one communication.

We have considered two different protocols for sending application data. The first one, which we have adopted in combination with LBR1, consists in the immediate forwarding of the received content to the $sinkBy_{i}$ node (Algorithm 4).

Alternatively, content can be buffered while it is received and be periodically sent piggybacked in leader messages. To do so, the $broadcast(LEADER,i,actualRound,sinkDistance_{i}+1)$ primitive must be redefined as $broadcast(LEADER,i,actualRound,sinkDistance_{i}+1,msgList_{i})$. This approach (Algorithm 5) is the one we have chosen in combination with LBR2.

**Algorithm 4:** Direct forwarding strategy executed by node $p_{i}$.
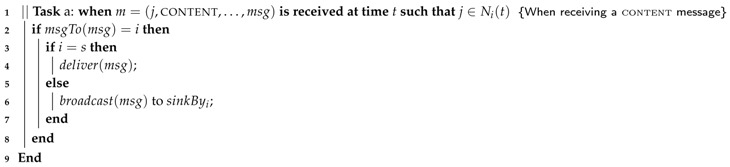


**Algorithm 5:** Piggybacking strategy executed by node $p_{i}$.
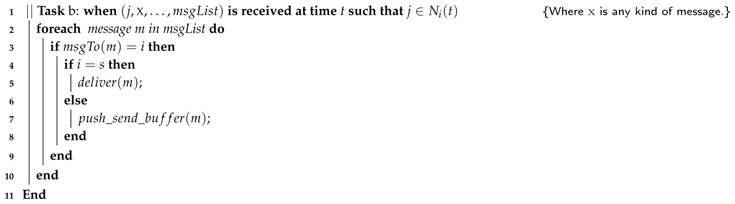


## 4. Evaluation

In this section, we describe an evaluation of the two versions of our approach, which have been simulated using Castalia [24] and OMNeT++ [25]. To have a comparative assessment, we introduce in the evaluation two reference protocols, AODV [20], particularly the implementation found in [26], and RASeR [21].

### 4.1. Simulation Environment

Castalia is a simulator based on OMNeT++ and commonly used for extracting performance metrics in WSNs [27].

Regarding the hardware configuration for Castalia, every simulation has been configured using the CC2420 radio with 0 dBm of transmission power. Additionally, Castalia provides a parameter called PLd0, which models the know path loss at a reference distance $d_{0}$. When using this parameter, Castalia provides the option of modifying the communication range [28]. The parameter PLd0 is calculated as follows:

Tx_power−max(receiver_sesitivity,noise_floor+5)−10×path_loss_exponent×log(range).

In our case, we have used the default 50 meters value for the range variable. However, since we consider communication collisions and interferences, the communication range is much lower than the nominal.

For the MAC layer, we have adopted the Carrier Sense Multiple Access with Collision Avoidance CSMA/CA protocol except for the case of RASeR, which imposes by default a Global TDMA MAC layer.

### 4.2. Experiment Description

We have carried out extensive experiments to evaluate the performance in terms of message delivery rate (We define message delivery rate as the number of CONTENT messages delivered to the sink divided by the total number of messages transmitted in the WSN.) and end-to-end delay.

Our main concerns in the evaluation are scalability and resiliency to node mobility. Henceforth, the relevant configuration parameters to feed the evaluations have been network size (number of nodes) and node mobility (maximum velocity).

We have defined a constant node density for all the tests, thus the network size determines the simulation area.

Every node of our simulations (including the sink node) is assumed to move according to a Random Walk mobility model [29]. A random direction and velocity, between 0 and the maximum velocity, is generated every second. This movement is divided in five sub-movements, i.e., one every 200 milliseconds, in order to effectively make a smooth movement.

A summary of the configuration parameters used for the experiments is shown in Table 1.

Preliminary evaluations of the algorithms LBR1 and LBR2 showed that timeout values should be slightly higher than the heartbeat period, which is 0.2 s. We do not define bounds in buffer size. Furthermore, results reported in [11] show that the best performances are obtained when messages are not acknowledged by the receiver.

In the original work of RASeR [21], a transmission range of 250 m is used, which we consider unrealistic for the kind of technology we used. Instead, in our simulations, we have assigned a common range for all the protocols.

Finally, in order to optimise the performance of the protocols, we have adjusted the length of the packet for each protocol. In the cases of LBR1 and AODV, we send a single content packet of 10 bytes, whereas, for LBR2 and RASeR, we send a list of ten messages increasing the packet size to 100 bytes.

### 4.3. Evaluation Results

#### 4.3.1. Delivery Rate

In order to compute the evaluation figures, data messages have been generated at the defined rate of 0.2 per second and its reception checked at the sink node. We have simulated a set of 120 configurations, each one being a combination of the parameters reported in the previous subsection. We have performed at least ten repetitions for every configuration. Variances obtained for each configuration are low, between 0.001 and 0.09, thus the figures presented here refer only to the averages of the results obtained.

**Figure 2:**
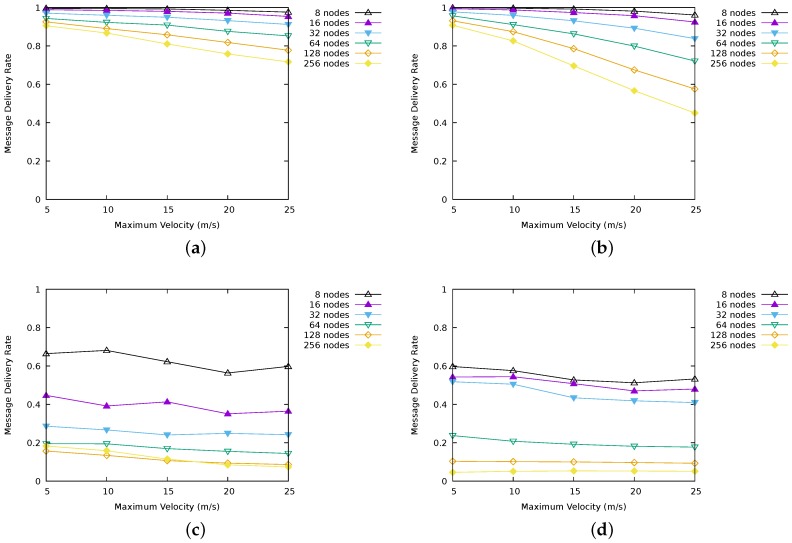
Message delivery rates for the simulated protocols. Results for a generation rate of four messages per node and second. (**a**) LBR1 message delivery rate; (**b**) LBR2 message delivery rate; (**c**) AODV message delivery rate; (**d**) RASeR message delivery rate.

**Figure 3:**
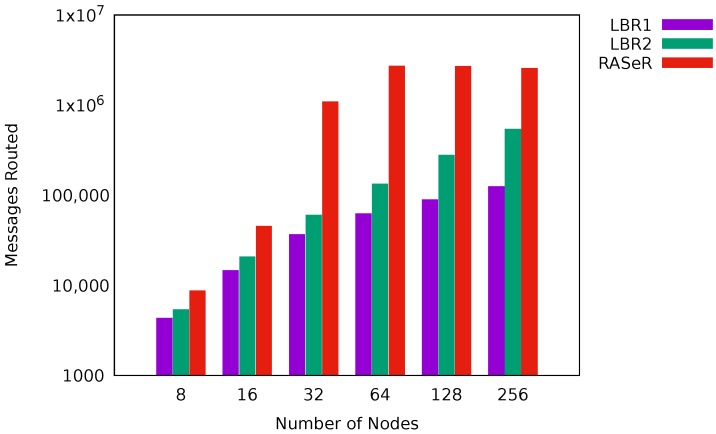
Number of messages routed towards the sink. Results for a maximum velocity of 20 m/s. Note the logarithmic scale on the *y*-axis.

Figure 2 illustrates the values of message delivery rates obtained for LBR1, LBR2, AODV and RASeR. LBR1 (Figure 2a) shows the best performance, followed by LBR2, which is negatively affected by high mobility. AODV (Figure 2c) presents low delivery rates in general, and poor scalability. On the other hand, RASeR provides a decent message delivery rate up to 32 nodes, obtaining nearly 60%. In this protocol, we have observed a problem related to message duplication, resulting in network congestion. Intuitively, global TDMA imposes an upper bound on the number of messages sent per cycle. Consequently, if the number of messages received is continuously higher than that upper bound, eventually it will cause a buffer overflow. This situation can be appreciated in Figure 3, which represents the overall number of messages that each protocol has used in order to route multi-hop communications towards the sink (i.e., excluding the first hop), a parameter with hight impact on energy consumption. Note that, despite LBR1, LBR2 and RASeR sharing the routing criteria based on the number of hops, RASeR is very inefficient regarding the number of generated messages.

**Figure 4:**
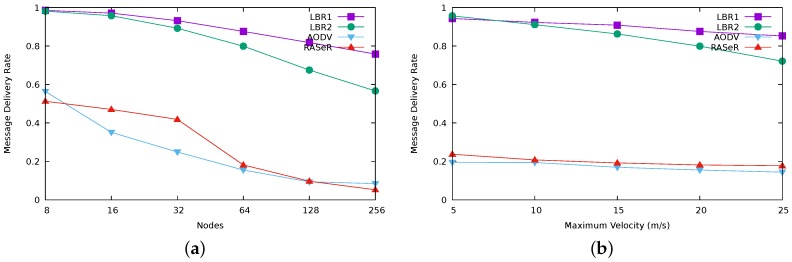
Comparative results for the delivery rate. (**a**) delivery rate regarding scalability; (**b**) delivery rate regarding mobility.

As a summary, Figure 4 illustrates some comparative results for the four protocols. Figure 4a compares delivery rate for a fixed maximum velocity of 20 m/s, and Figure 4b compares delivery rate for a network size of 64 nodes.

**Figure 5:**
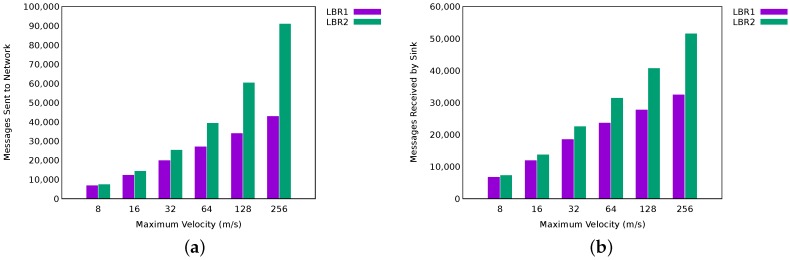
Number of messages sent and received by LBR1 and LBR2 for a maximum velocity of 20 m/s. (**a**) messages sent to network; (**b**) messages received by the sink.

It should be noted that the delivery rate figures for LBR1 and LBR2 benefit in part from the fact of disabling communications, since messages not transmitted do not account in total (We are assuming that a not sent message is managed, and possibly re-sent as new, by the application layer.). Since LBR1 is more reactive than LBR2, this mechanism is activated more frequently in the former. Figure 5a shows that effectively LBR1 has sent much less number of messages than LBR2. This is beneficial from an energy efficiency point of view with a positive impact on energy consumption. However, LBR2 is more effective since it delivers a higher absolute number of messages to the sink (Figure 5b).

**Figure 6:**
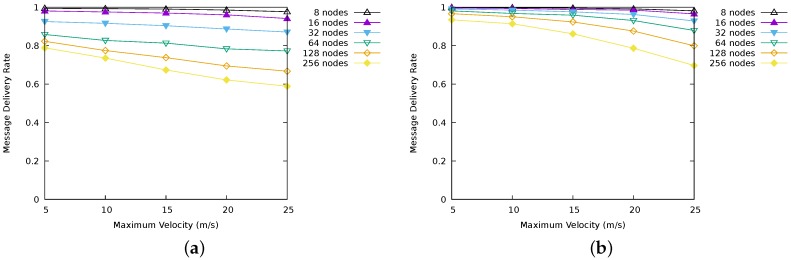
Message delivery rate for LBR1 and LBR2. Results for a generation rate of 10 messages per node and second. (**a**) LBR1 message delivery rate; (**b**) LBR2 message delivery rate.

Recall that, for all the above experiments, we have used a message generation rate of four messages per second and node. During the simulations, we have observed that, in the case of LBR2, the message payload was not completely used. Thus, we have carried out a new series of experiments increasing the message generation rate to 10 messages per second and node. The results can be seen in Figure 6. As shown in Figure 6a,b, LBR2 performs better than LBR1 as the rate of message generation increases. While LBR1 has to route each message individually, increasing network congestion, LBR2 is able to take advantage of its ability to send multiple messages in a single packet, increasing the message delivery rate.

#### 4.3.2. End-to-End Delay

**Figure 7:**
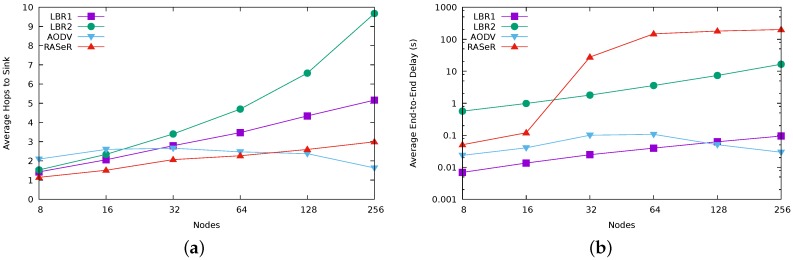
End-to-end delay results about scalability (network size). Results for a maximum velocity of 20 m/s. Note the logarithmic scale on the *y* axis for Figure 7b. (**a**) hops to reach the sink (average); (**b**) end-to-end delay (average).

**Figure 8:**
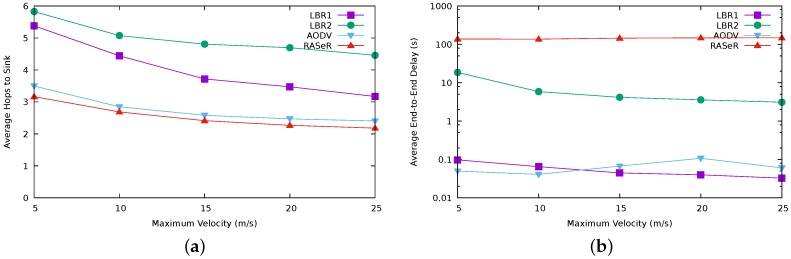
End-to-end delay results about resilience to mobility. Results obtained for 64 nodes. Note the logarithmic scale on the *y* axis for Figure 8b. (**a**) Hops to reach the sink (average); (**b**) End-to-end delay (average).

We have also measured the end-to-end delay and the number of hops. Figure 7 shows the results for a velocity of 20 m/s. For the same parameters, Figure 8 summarizes the results regarding mobility for the particular case of 64 nodes. Recall that in LBR1 and (specially) in LBR2, a node only sends messages when it is connected, which results in high figures for these protocols for the number of hops. On the other hand, LBR2 exhibits higher latencies than LBR1 due to its lazy forwarding strategy.

Figure 7b highlights again the performance fall in RASeR for a size greater than 32 nodes, as we have previously identified (Figure 4a).

## 5. Conclusions

We have presented a routing approach for mobile wireless sensor networks, which is based on the election of a leader node and the construction of a spanning tree with the root in the sink node, which is mobile as well. On the basis of local information about link quality, leader elections are performed in order to restructure the spanning tree so that message delivery rates could be optimised.

We have designed two protocols to implement the approach, LBR1, and LBR2. Both protocols use a heartbeat mechanism to react to node mobility. However, while LBR1 is fully reactive, in the sense that leader-election messages are forwarded immediately providing a good delivery rate, LBR2 buffers the messages to be forwarded periodically, resulting in a more effective performance at the price of higher delays.

We have simulated our protocols in Castalia and carried out comparative evaluations with respect to two other MWSN protocols, AODV and RASeR, to determine the behaviour of the protocols with respect to their scalability and their resilience to mobility, measured as the node velocity. In general, our protocols outperform the other two.

Although we have not identified a specific application scenario for our approach, evaluation results show that it can be applied to MWSN including hundreds of elements that move freely in areas of a size in the order of at least tens of hectares, with velocities of up to 25 m/s. This includes practical applications in smart cities or person and animal tracking, among others.

The approach has the potential for great scalability. Since, in our protocols, a node is conscious of its connectivity and only sends messages when it is connected, message traffic could be optimised by using buffering and opportunistic communication. In order to apply to a specific scenario, our approach brings a combination of possibilities for finding the appropriated configurations that fits the trade-offs among the different parameters, such as protocol version (LBR1 or LBR2), heartbeat frequency (which has a high impact on reaction times and henceforth on mobility resilience), transmission range, or packet size.

For future work, we will also focus on extensions of the approach oriented to the use of multiple sinks and the combination of static and dynamic elements, in order to face high-scale problems.

## Figures and Tables

**Table 1:** Simulation parameters.

Parameters	Values
Buffer discharge time (seconds)	20
Number of nodes	8, 16, 32, 64, 128, 256
Node density (nodes per square meter)	0.003
Transmission range, *r* (meters)	50
Maximum node velocity (meters per seconds)	5, 10, 15, 20, 25
Message generation rate per node (messages per second)	4 (for most simulations), 10
Packet size (bytes)	10 for AODV and LBR1; 100 for RASeR and LBR2
Simulated interval (seconds)	600
